# Endothelial Glycocalyx Shedding Occurs during Ex Vivo Lung Perfusion: A Pilot Study

**DOI:** 10.1155/2019/6748242

**Published:** 2019-08-25

**Authors:** Timothy M. Sladden, Stephanie Yerkovich, Douglas Wall, Maxine Tan, William Hunt, Jonathan Hill, Ian Smith, Peter Hopkins, Daniel C. Chambers

**Affiliations:** ^1^Queensland Lung Transplant Service, The Prince Charles Hospital, Brisbane 4032, Australia; ^2^Faculty of Medicine, The University of Queensland, Brisbane, Australia; ^3^Department of Cardiothoracic Surgery, The Prince Charles Hospital, Brisbane 4032, Australia; ^4^Perfusion Services, The Prince Charles Hospital, Brisbane 4032, Australia; ^5^School of Veterinary Science, The University of Queensland, Gatton 4343, Australia; ^6^Department of Anaesthetics, The Prince Charles Hospital, Brisbane 4032, Australia

## Abstract

**Background:**

Damage to the endothelium has been established as a key pathological process in lung transplantation and ex vivo lung perfusion (EVLP), a new technology that provides a platform for the assessment of injured donor lungs. Damage to the lung endothelial glycocalyx, a structure that lines the endothelium and is integral to vascular barrier function, has been associated with lung dysfunction. We hypothesised that endothelial glycocalyx shedding occurs during EVLP and aimed to establish a porcine model to investigate the mechanism underlying glycocalyx breakdown during EVLP.

**Methods:**

Concentrations of endothelial glycocalyx breakdown products, syndecan-1, hyaluronan, heparan sulphate, and CD44, were measured using the ELISA and matrix metalloproteinase (MMP) activity by zymography in the perfusate of both human (*n* = 9) and porcine (*n* = 4) lungs undergoing EVLP. Porcine lungs underwent prolonged EVLP (up to 12 hours) with perfusion and ventilation parameters recorded hourly.

**Results:**

During human EVLP, endothelial glycocalyx breakdown products in the perfusate increased over time. Increasing MMP-2 activity over time was positively correlated with levels of syndecan-1 (*r* = 0.886; *p*=0.03) and hyaluronan (*r* = 0.943; *p*=0.02). In the porcine EVLP model, hyaluronan was the only glycocalyx product detectable during EVLP (1 hr: 19 (13–84) vs 12 hr: 143 (109–264) ng/ml; *p*=0.13). Porcine hyaluronan was associated with MMP-9 activity (*r* = 0.83; *p*=0.02) and also with dynamic compliance (*r* = 0.57; *p*=0.03).

**Conclusion:**

Endothelial glycocalyx products accumulate during both porcine and human EVLP, and this accumulation parallels an accumulation of matrix-degrading enzyme activity. Preliminary evidence in our porcine EVLP model suggests that shedding may be related to organ function, thus warranting additional study.

## 1. Introduction

Ex vivo lung perfusion (EVLP) is a new technology designed to improve lung function in retrieved organs with the objective to increase utilisation and decrease acute posttransplant dysfunction [1, 2]. EVLP is a process whereby lungs are perfused with a hyperoncotic solution at normothermia, with concurrent ventilation, providing a platform for lung assessment and reconditioning. Whilst undergoing EVLP, failing lungs show impaired ventilation [3] and perfusion [4] because of dysfunction of the endothelial [5, 6] and alveolar epithelial [7–10] barriers, although the mechanism remains incompletely understood. The endothelium is the first barrier to fluid extravasation with transplant studies associating endothelial injury with deleterious outcomes such as alveolar oedema, perivascular fluid accumulation, and endothelial thrombosis [11, 12]. Increased perfusate levels of endothelial activation biomarkers during EVLP are associated with the development of primary graft dysfunction after transplant [10]. Furthermore, syndecan-1, a biomarker of endothelial glycocalyx injury, has been shown to accumulate in the EVLP perfusate with decreased concentrations associated with suitability for transplantation [13].

**Figure 1:**
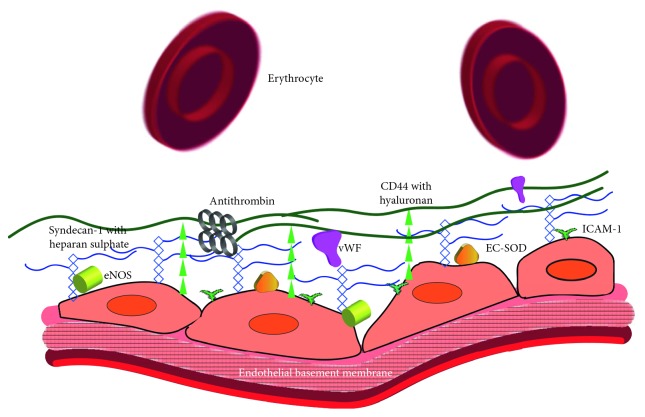
Endothelial glycocalyx: longitudinal cross section of blood vessels with endothelial cells adherent to the endothelial basement membrane. The endothelial glycocalyx is secreted by the underlying endothelial cells and projects 0.5–1.2 *μ*m into the blood vessel lumen forming a negatively charged meshwork of glycosaminoglycan branches that interact and form a barrier to the overlying albumin, macromolecules, and red blood cells alike. The most numerous components of the glycocalyx are the anchoring proteoglycan syndecan-1 with its attached glycosaminoglycans, namely, heparan sulphate (represented by the blue rectangles and lines) and chondroitin sulphate (not drawn). Another structural component is hyaluronan, which occurs in chains, of several million dalton in size, attached to the underlying endothelium by the glycoprotein CD44 (represented by the green triangles and lines). The glycocalyx interacts with other endothelial structures such as intracellular adhesion molecule-1 (ICAM-1; represented by the green T's) by acting as a physical barrier and preventing leucocytes from reaching these much shorter glycoprotein adhesion molecules. Shown also are the adherent plasma proteins that interact with the glycocalyx, providing vital endothelial functions such as haemostasis by binding von Willebrand factor (vWF; represented by the magenta objects) and antithrombin (represented by the grey circles). In addition, binding extracellular superoxide dismutase (EC-SOD; represented by the gold triangles) and endothelial nitric oxide synthase (eNOS; represented by the yellowish green cylinders), the glycocalyx contributes to protection from free radical injury and mechanotransduction, respectively.

The endothelial glycocalyx (Figure 1) coats the luminal surface of all blood vessels and is composed of glycosaminoglycans (GAGs), namely, heparan sulphate, chondroitin sulphate, and hyaluronan, anchored to the underlying endothelium via proteoglycans such as syndecan-1 and glycoproteins including CD44 [14, 15]. Damage to the glycocalyx can be caused by several processes common in the transplantation pathway including hypotension/shock [16, 17] and ischaemic reperfusion injury [18, 19]. The mechanism(s) leading to endothelial glycocalyx breakdown and shedding are still emerging; however, it is known that free radicals created during ischaemic reperfusion injury [20, 21], along with the subsequent activation of matrix metalloproteinases [20, 22] and cell surface endoglycosidases [23–25], can all shed different components of the endothelial glycocalyx [26]. In addition, lung transplant studies have demonstrated MMP activity to be associated with graft dysfunction on ex vivo lung perfusion [27] and with ischaemic reperfusion injury in recipients [28, 29].

Given the integral role the endothelial glycocalyx plays in both mechanotransduction and vascular barrier function, we hypothesised that endothelial glycocalyx integrity may be important for organ performance during EVLP. No studies have investigated the relationship between endothelial glycocalyx breakdown and organ dysfunction on EVLP. The aims of this study were (1) to determine if multiple endothelial glycocalyx breakdown products are shed into the perfusate of human lungs on EVLP using previously validated biomarkers, (2) to investigate the mechanism(s) behind this shedding in EVLP by the establishment of a porcine ex vivo lung perfusion model, and (3) to correlate increases in endothelial glycocalyx biomarker concentrations with shedding of the glycocalyx visualised utilising sidestream dark field imaging.

## 2. Methods

### 2.1. Ethical Approval

Human EVLP experiments were approved by The Prince Charles Hospital Human Research and Ethics Committee (HREC/13/QPCH/154). Samples were accessed when the donor family had consented to research as part of the consent process for organ donation. The porcine EVLP studies were approved by the Animal Ethics Committee, The University of Queensland, Australia. Pigs used in this study were treated in accordance with the *Animal Care and Protection Act 2001*, *Queensland*, and the *Australian code for the care and use of animals for scientific purposes* published by the National Health and Medical Research Council, Australia. All pigs were sourced from The University of Queensland Gatton campus' commercial piggery.

### 2.2. Human Ex Vivo Lung Perfusate Samples

Human EVLP perfusate samples were derived from 9 human lung perfusion cases (5 clinical and 4 experimental; Table 1). Organ retrieval and human lung ex vivo lung perfusion were performed under standard protocols at The Prince Charles Hospital (Supplementary [Supplementary-material supplementary-material-1]). Of the 9 lungs, only 3 were deemed suitable for transplant using clinical criteria (detailed in Supplementary [Supplementary-material supplementary-material-1]).

The perfusate samples were centrifuged (5000*g* for 10 mins), and the supernatant was stored at −80°C. The samples were collected at the start and then every 15 minutes, with a minimum of 3 samples available.

In the experimental group of 4 lungs, the initial samples were not available from the start of perfusion (time on rig before samples were collected is noted in Table 1) because of ethical constraints that limited the gathering of samples whilst the organs were still undergoing clinical EVLP. After being deemed not suitable for transplant, the experimental EVLP lungs underwent prolonged perfusion (60–90 min) as part of another study. In the experimental group lactate, glucose and PaO_2_ were measured from blood leaving the left atrial remnant. No lung perfusion and ventilation data (dynamic compliance, pulmonary vascular resistance, pulmonary artery pressure, and flow rate) was available.

### 2.3. Establishment of Porcine Protocol

To define the role of endothelial glycocalyx breakdown and lung function, we established a porcine model of prolonged EVLP and aimed to measure endothelial glycocalyx breakdown products in the perfusate. The porcine EVLP protocol is included in Supplementary [Supplementary-material supplementary-material-1] detailing the retrieval technique, setup of the ex vivo lung perfusion system, and reperfusion and ventilation strategy. Four healthy (40–48 kg) pigs were anaesthetized and surgically prepared for lung retrieval before death was induced by intracardiac injection of pentobarbital (Virbac, Australia), resulting in ventricular fibrillation with organs immediately retrieved as per human lung retrieval. After 4 hrs in static cold storage, the lungs were connected to the open atrium Vivoline LS1 EVLP rig (Vivoline Medical, Sweden). In a similar method to that described by Cypel et al. [30], the lungs were perfused with a Steen solution with autologous blood and gradually warmed (see Supplementary [Supplementary-material supplementary-material-1]). Once perfusate temperature reached 32°C, ventilation was commenced. Perfusion and ventilation were continued for 12 hours or until lungs were too oedematous to continue EVLP, as indicated by the perfusate clearly accumulating in the tracheal tube.

### 2.4. Porcine Ex Vivo Lung Perfusion Evaluation

Sampling and recording of perfusion and ventilation parameters were undertaken hourly on EVLP. Gas exchange function was measured using arterial blood gases (i-STAT, Abbott Point of Care, USA). Perfusate samples were collected for glucose and endothelial glycocalyx breakdown product analysis (10 ml was centrifuged, and the supernatant was stored at −80°C for later batch analysis). Biopsies of lung tissue were collected at 3 hrs and at completion for wet : dry weight ratio analysis.

### 2.5. Measurement of Endothelial Glycocalyx Breakdown Products

Human endothelial glycocalyx breakdown products were measured in the perfusate using enzyme-linked immunosorbent assay (ELISA) kits for hyaluronan, syndecan-1, CD44 (R&D Systems Inc., Minneapolis, MN, USA), and heparan sulphate (Cusabio Biotech, Wuhan, China) following the manufacturer's instructions.

Endothelial glycocalyx breakdown products in the porcine EVLP perfusate were measured using ELISA kits for hyaluronan (R&D Systems Inc., Minneapolis, MN, USA) and heparan sulphate (Cusabio Biotech, Wuhan, China). In addition, porcine heparan sulphate proteoglycan (Elabscience Biotechnology, Wuhan, Hebei, China), heparan sulphate (MyBioSource Inc., CA, USA), and porcine syndecan-1 (Cusabio Biotech, Wuhan, China) ELISA kits were utilised in an attempt to measure endothelial glycocalyx breakdown products in the porcine EVLP perfusate.

### 2.6. Matrix Metalloproteinase Activity

To investigate the pathophysiology underlying endothelial glycocalyx dysfunction during EVLP, levels of matrix metalloproteinase 2 and 9 activity were determined. Perfusate samples of both human and porcine lungs were subjected to electrophoresis (130 volts for 120 minutes with molecular weight standards) followed by gel zymography (Novex 10% zymogram gel (0.1% gelatin), Invitrogen) according to the manufacturer's instructions. Stained gels, which showed zones of lysis as clear areas against a blue background, were then photographed, and images were analyzed using ImageJ (US National Institutes of Health, USA) to calculate relative area.

### 2.7. Porcine Lung Imaging Using Sidestream Dark Field Microscopy

To visually correlate endothelial glycocalyx shedding, pulmonary pleural capillaries were visualized by sidestream dark field (SDF) microscopy, a technique validated in human sublingual [31] and renal [32] capillary glycocalyx studies. Similar to the technique outlined by den Uil et al. [33] for pulmonary pleural and alveolar capillaries, an SDF Microscan video microscope (INOVANZ, Australia) was used to obtain two-dimensional video images (at 25 Hz) of pulmonary structures. The detailed methods for this technique are described in Supplementary [Supplementary-material supplementary-material-1] with video images captured in situ before lungs were retrieved and once lungs were stable on the EVLP circuit and fully ventilated at the 1, 6, and 12 hr (or completion) time points.

### 2.8. Statistical Analysis

Results are expressed as median (interquartile range) unless otherwise stated. All analysis was performed using Prism v7 (GraphPad Software Inc., CA, USA). Correlations were assessed using the Spearman rank-order correlation test. The Wilcoxon signed-rank test was used to assess the change in biomarker levels from start to completion of perfusion for both human and porcine lungs. The Mann–Whitney test was used to assess differences between transplanted and nontransplanted lungs.

## 3. Results

### 3.1. Endothelial Glycocalyx Breakdown Products Accumulate in Human Ex Vivo Lung Perfusate

**Figure 2:**
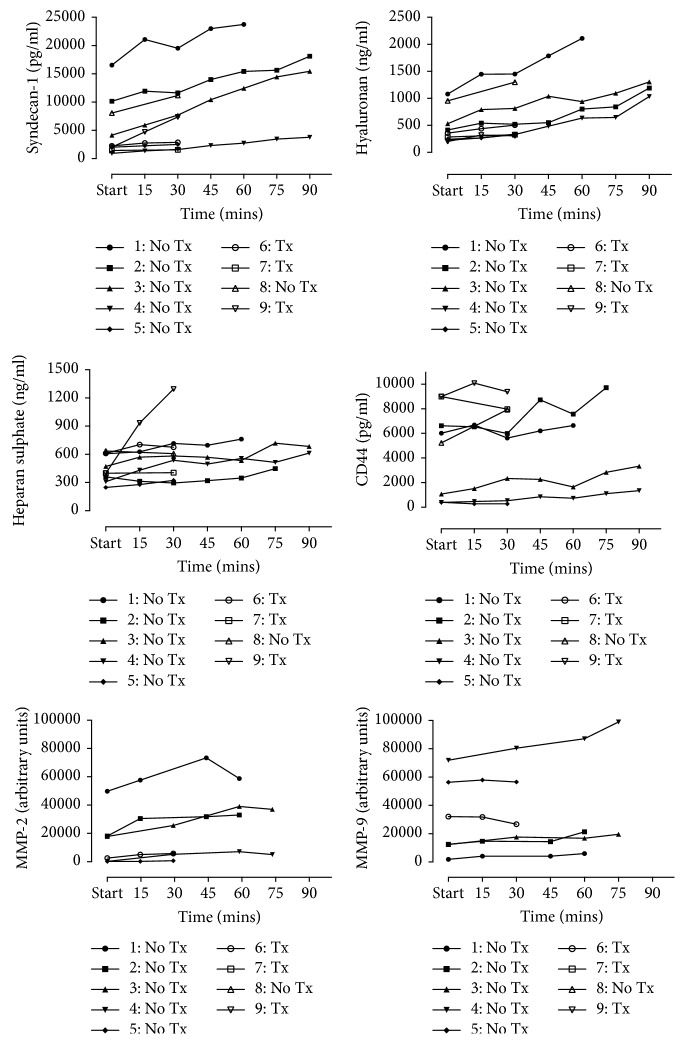
Perfusate levels of endothelial glycocalyx breakdown products and matrix metalloproteinases (MMPs) over time during human ex vivo lung perfusion (EVLP). The start time point varied for lungs and is detailed in Table 1. Lungs 1–4 were experimental lungs that underwent prolonged perfusion. Tx: lungs transplanted after EVLP; No Tx: lungs not transplanted after EVLP; pg/ml: pictograms per ml; ng/ml: nanograms per ml.

**Figure 3:**
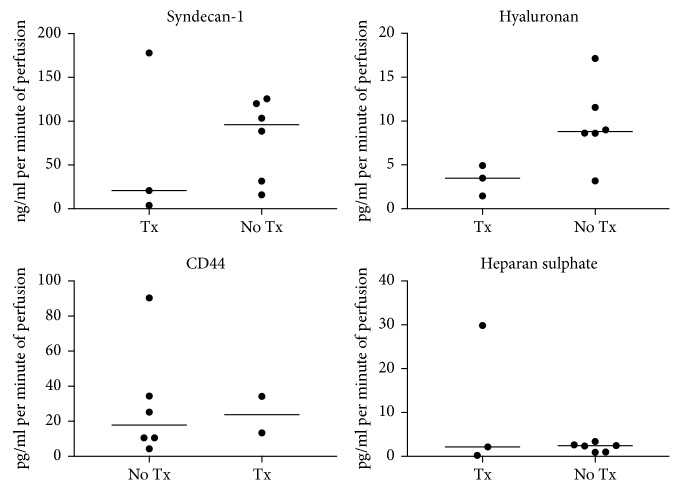
Rate of accumulation of perfusate endothelial glycocalyx breakdown products by outcomes (transplanted (Tx) vs not transplanted (No Tx)). None of the differences are statistically significant.

Endothelial glycocalyx breakdown products accumulate in the perfusate from start to completion with significant increases in syndecan-1 (2259 (1740–9107) to 7368 (2693–16786) pg/ml; *p*=0.004), hyaluronan (257 (233–741) to 1033 (333–1301) ng/ml; *p*=0.004), heparan sulphate (399 (334–608) to 612 (426–723) ng/ml; *p*=0.012), and MMP-2 (10145 (2333–26005) to 19430 (3991–42447) arbitrary units (AU); *p*=0.031) (Figure 2). Although limited by small numbers, there was a nonsignificant trend for increased levels of hyaluronan and syndecan-1 in organs that were not transplanted compared to transplanted lungs (Figure 3).

### 3.2. Endothelial Glycocalyx Breakdown Is Associated with MMP Activity

The relationships between endothelial glycocalyx breakdown products and one of their key degradation enzymes, matrix metalloproteinase, were assessed at completion of EVLP. Notably, there was a strong positive relationship between MMP-2 activity, but not MMP-9, and increased syndecan-1 (*r* = 0.886; *p*=0.03) and hyaluronan (*r* = 0.943; *p*=0.02) levels. No significant relationship existed between the accumulation of hyaluronan and its main endothelial binding receptor CD44 (*r* = −0.214; *p*=0.62), suggesting increased hyaluronan was likely related to fragmentation of high-molecular-weight hyaluronan rather than en bloc endothelial shedding. No association was seen between heparan sulphate and its main binding proteoglycan syndecan-1 (*r* = 0.517; *p*=0.161) although syndecan-1 and hyaluronan levels were closely related (*r* = 0.783; *p*=0.017).

### 3.3. Hyaluronan Accumulates in Porcine EVLP Perfusate and Is Associated with MMP Activity and Lung Function

**Figure 4:**
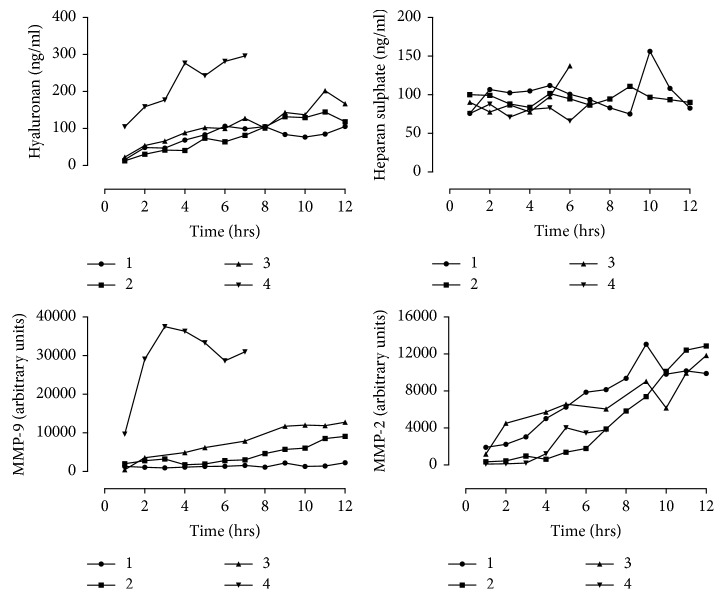
Porcine EVLP perfusate endothelial glycocalyx breakdown products and MMP levels over time.

There was a trend for increased hyaluronan accumulation in the EVLP perfusate over time (19 (13–84) vs 143 (109–264) ng/ml; *p*=0.13), whilst heparan sulphate levels remained stable (83 (75–97) vs 89 (84–126) ng/ml; *p*=0.38) (Figure 4). The lack of increase of heparan sulphate in the perfusate over time may have been related to sensitivity of the assay as levels were at the lower limit of detectability. The endothelial glycocalyx markers heparan sulphate proteoglycan and syndecan-1 were below the level of detection (data not shown). A nonsignificant increase was seen for MMP-9 and MMP-2 activity over perfusion, as shown in Figure 4 (*p*=0.13 and *p*=0.13, respectively). There was a negative association between hyaluronan and dynamic compliance (*r* = −0.74; *p*=0.05), at the start and completion of EVLP perfusion. There was also a significant strong positive association between hyaluronan and MMP-9 (*r* = 0.83; *p*=0.02), but the association with MMP-2 (*r* = 0.67; *p*=0.08) did not reach significance.

**Figure 5:**
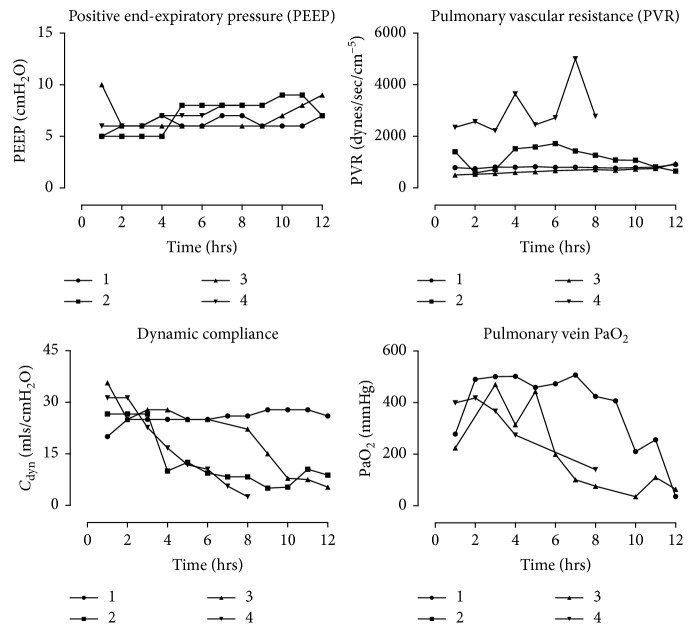
Porcine EVLP and ventilation parameters first recorded at 1 hr after the initiation of perfusion. Pulmonary vein sampling of PaO_2_ was collected from blood leaving the left atrial remnant with FiO_2_ 100%.

We also assessed ventilation and perfusion parameters whilst porcine lungs underwent EVLP. Initially, pulmonary vascular resistance was extremely high in all 4 porcine lungs before rapidly decreasing over the first hour as lungs were warmed to 37°C and increased to full flow (Figure 5). Subsequently, pulmonary vascular resistance slowly increased over perfusion after the first 1 to 2 hrs. In all lungs, there was a trend towards decreased pulmonary function with decreased PaO_2_ and lung compliance over perfusion (Figure 5). Positive end-expiratory pressure was gradually increased over the time course as the level of interstitial oedema increased. The wet : dry weight ratio increased from 4.6 (3.8–6.2) at the 3 h to 7.1 (5.9–8.9) at completion.

### 3.4. Porcine EVLP Is Comparable to Human EVLP

**Figure 6:**
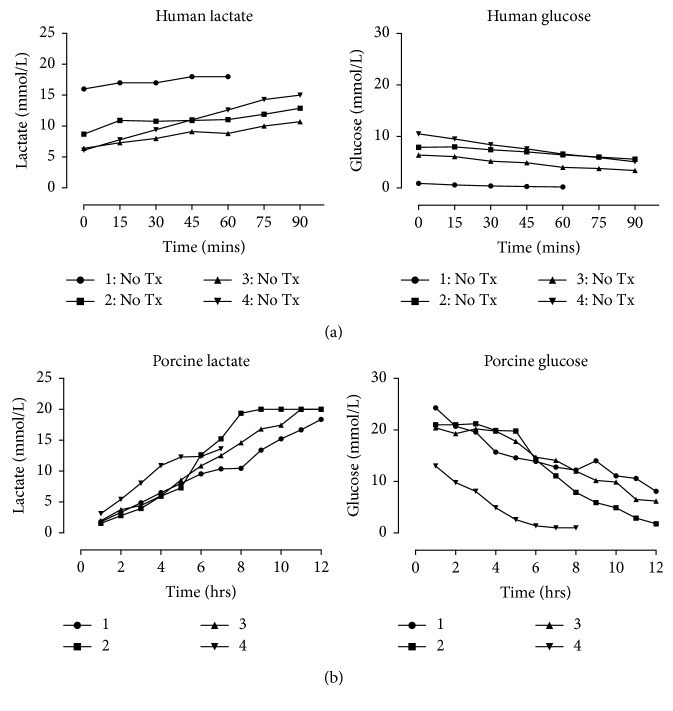
(a) Human and (b) porcine EVLP perfusate glucose and lactate levels.

In both human and porcine EVLP, glucose was metabolised over time with a subsequent increase in lactate levels detectable in the EVLP perfusate (Figures 6(a) and 6(b)). Glucose utilisation rates were similar between human and porcine lungs (0.029 (0.015–0.053) vs 0.025 (0.020–0.027) mmol/L/min; *p*=0.68); however, rates of lactate accumulation were significantly higher in the human perfusate (0.047 (0.037–0.086) vs 0.024 (0.017–0.025) mmol/L/min; *p*=0.03). Rates of hyaluronan accumulation were significantly higher in human lungs than in porcine lungs (8.61 (3.3–10.27) vs 0.173 (0.13–0.392) ng/ml/min; *p*=0.002). MMP-2 was significantly higher in the human lung perfusate (130.7 (44.28–173.9) vs 12.95 (9.37–16.75) AU/ml/min; *p*=0.019), with a similar pattern for MMP-9 (80.23 (37.3–199) vs 13.47 (3.46–42.36) AU/ml/min; *p*=0.11) that did not reach significance.

### 3.5. Sidestream Dark Field Microscopy Was Unable to Measure Endothelial Glycocalyx Shedding

**Figure 7:**
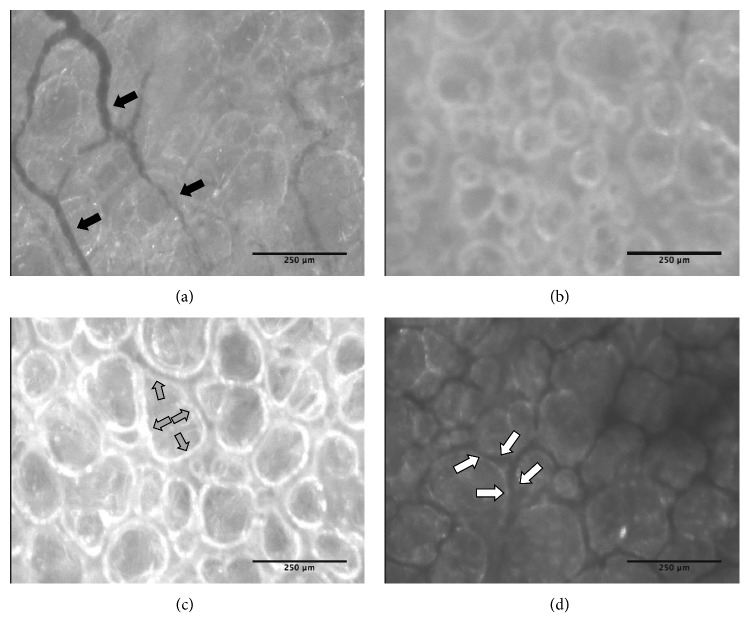
Porcine sidestream dark field imaging. Representative images are captured frames from a video recorded using the ImageJ software. (a) In situ normal pleural blood vessels before organ retrieval (black arrows). (b) In situ normal alveoli before organ retrieval. (c) Alveoli on EVLP in healthy, well-ventilated nonoedematous portions of the lung with the grey arrows inside an alveolus indicating the margin. (d) Alveoli on EVLP in haemorrhagic oedematous portions of the lung with the white arrows highlighting the interalveolar septum that contains haemoglobin giving it the black discolouration. Scale bar is 250 micrometers.

Utilising the currently available technology, the SDF microscope was unable to capture images of pulmonary pleural capillaries of sufficient quality to visualise the glycocalyx because of movement artifacts from the heart. The glycocalyx of visceral pleural capillaries was able to be visualised as shown in representative images taken from the captured video of lungs *in situ* (Figure 7(a)). On EVLP, the visceral pleural vessels were absent, suggesting that there was no perfusion to the pleura. Because of these limitations, we were unable to correlate accumulation of the endothelial glycocalyx breakdown products in the perfusate with direct visualisation of shedding of the glycocalyx. The SDF imaging allowed us to clearly visualize the alveoli while on extended EVLP (Figure 7(b)); however, there was no difference in the lung images recorded at 1, 6, and 12 hrs (or completion) (data not shown). There was a marked difference in the appearance of oedematous areas, hemorrhagic lung tissue (Figure 7(d)), and normal ventilated lung tissue (Figure 7(c)) on SDF imaging, taken at peak of inspiration, which correlated with visual inspection.

## 4. Discussion

This study demonstrated that endothelial glycocalyx breakdown products accumulate in the human EVLP perfusate. Furthermore, in our porcine EVLP model, hyaluronan in the perfusate was negatively related to lung function, with increased MMP activity being demonstrated in both human and porcine EVLP perfusate. Our findings provide preliminary evidence that shedding of the endothelial glycocalyx is occurring in EVLP and suggest it may have a role in lung function on EVLP. The importance of the endothelial glycocalyx in pulmonary vascular integrity has been demonstrated in mouse studies with shedding of the endothelial glycocalyx resulting in neutrophil adhesion [34], perfusion failure of microvessels, increased alveolar septal width, and pulmonary artery pressures [35]. Further evidence arises from mouse studies associating ischaemic reperfusion with injury to the glycocalyx and shedding of pulmonary endothelial syndecan-1 leading to increased vessel permeability and stress fiber formation [36]. The accumulation of endothelial glycocalyx breakdown products during trauma in the systemic circulation of humans [37, 38] suggests that similar glycocalyx dysfunction may occur in EVLP.

Evidence supporting a possible role of endothelial glycocalyx dysfunction in poor organ performance during EVLP comes from the observed association between endothelial glycocalyx breakdown products and their known degradation enzymes [26]. We studied gelatinases (MMP-2 and MMP-9), as these have previously been associated with graft dysfunction on ex vivo lung perfusion [27], ischaemic reperfusion injury [28, 29], and rejection in lung transplantation [39]. Furthermore, MMP-2 is constitutively expressed by pulmonary endothelial cells and fibroblasts, whilst MMP-9 is associated with inflammatory cells [40]. Using the porcine lung EVLP model, Soccal et al. [27]correlated bronchoalveolar lavage MMP-2 and MMP-9 activity with altered alveolar-capillary permeability and attributed this to injury of the extracellular matrix and neutrophil influx. Whilst Andreasson et al. confirmed this accumulation of MMPs in human EVLP perfusate [13]. Recent studies into endothelial glycocalyx pathophysiology have demonstrated a clear association between MMP activity and shedding of syndecans from the glycocalyx [41–43]. Our study results support a pathological role of MMPs in EVLP and suggest a relationship between MMP enzyme activity and endothelial glycocalyx shedding.

Shedding of endothelial glycocalyx is a complex process with multiple pathways [44]. Enzymes such as heparanase and hyaluronidase derived from endothelial cells and platelets are activated in ischaemia reperfusion injury and cause shedding of heparan sulphate and hyaluronan, respectively [26]. We were not able to measure heparinase as heparin, a potent antiheparanase molecule, was included in our perfusate. In addition to enzymatic degradation, fragmentation of the glycocalyx can be caused directly by reactive oxygen species [45, 46]. Hyaluronan fragments have been demonstrated to precipitate MMP secretion and activation [47] along with stimulation of a sterile inflammatory response through toll-like receptor activation [48, 49]. Hyaluronan accumulation in human EVLP was associated with MMP-2 activity, whilst in porcine EVLP, it was associated with MMP-9. The difference may be related to different blood products utilised in the ex vivo lung perfusate. For human EVLP, leukocyte-depleted packed red blood cells that had been washed and irradiated were utilised, whilst in porcine EVLP, packed red blood cells were used, which contain more leucocytes that are known to produce MMP-9. Further research is needed to delineate the role of individual enzymes and how these relate to lung ischaemia reperfusion, glycocalyx injury, and organ function during EVLP.

The findings of this study suggest an association between endothelial glycocalyx shedding and lung dysfunction during EVLP. Other investigators have demonstrated that degradation of hyaluronan in experimental vascular studies leads to decreased shear-induced release of endothelial nitric oxide [50] and increased capillary permeability [51]. Our porcine model demonstrated hyaluronan accumulation was associated with decreased lung compliance and a corresponding increase in wet : dry weight ratio. Therapeutic high-molecular-weight hyaluronan has been shown to mitigate pulmonary hyperpermeability in lipopolysaccharide and ischaemic reperfusion injury mouse models, suggesting a critical role of hyaluronan in the lung endothelial glycocalyx [21, 52]. More work is needed to clarify the association between decreased lung compliance and hyaluronan seen in our study.

The porcine EVLP model in this study was performed similarly to existing models of prolonged open atrium porcine EVLP, with a gradual decrease in dynamic compliance, corresponding to increase pulmonary oedema [4, 53, 54]. Additionally, the changes in lactate and glucose are consistent with human EVLP studies [55, 56]. Although our porcine model appeared to mirror metabolism in human lungs maintained on EVLP, the only measurable glycocalyx breakdown product was hyaluronan with a concentration 20 times less than that observed in human lungs. Plausible explanations for this include the use of lungs from juvenile pigs, which may not have yet developed sufficient connective tissue composition [57]. Alternatively, the lack of endothelial glycocalyx breakdown products during porcine EVLP could have been related to the comparatively low pulmonary artery flow rate, as flow rate was only 40% of cardiac output (compared to 100% in human EVLP), resulting in lower shear stress on the endothelial wall [58]. Given the endothelial glycocalyx composition and its relatively fragile nature, processing techniques utilised in traditional histological evaluation of tissues result in destruction of the glycocalyx. The gold standard for functional glycocalyx imaging is intravital microscopy, utilising fluorescent labelled dextran molecules of varying size to give real-time images; however, this is limited to small animal models [59]. Although we were unable to utilise SDF imaging, the loss of pleural perfusion is a novel finding and is likely associated with the loss of bronchial blood vessels at retrieval [60]. The use of porcine lungs as a model for human EVLP is accepted, with this model performing similarly to those in the published literature; however, further work is needed to optimise investigative techniques for studying the endothelial glycocalyx.

We recognise several limitations to this study: Firstly, the low number of clinical EVLP runs performed at our center prevents us from drawing any firm conclusions regarding the utility of measuring glycocalyx products as biomarkers of organ function. We postulate that, with a large sample size, endothelial glycocalyx biomarkers could prove to be accurate markers of pulmonary endothelial health and valuable tools for organ selection. Secondly, similar to all studies utilising biomarkers to estimate glycocalyx injury, we were unable to quantify the contribution of the extracellular matrix constituents hyaluronan and heparan sulphate to levels in the perfusate [61]. However, unlike other studies which have examined endothelial glycocalyx breakdown products in the peripheral blood [37, 62, 63], we can exclude glycocalyx breakdown contributions from the systemic vasculature. In addition, the potentially confounding effects of metabolism of endothelial glycocalyx components can be excluded as they occur primarily in the liver and kidney [64]. Additional studies, combining direct imaging, biomarkers, and functional parameters, are needed to delineate the relationship between endothelial glycocalyx dysfunction and EVLP.

## 5. Conclusion

This study demonstrated that endothelial glycocalyx products can be measured in the human EVLP perfusate, raising the possibility of these being used as biomarkers for lung function and organ selection on EVLP. Although the findings of this pilot study require further investigation, the observed association between glycocalyx shedding and MMP activity may provide insight into the mechanisms underlying glycocalyx shedding during EVLP. This research highlights EVLP as a modality for evaluating the pulmonary endothelial glycocalyx and provides a platform for future investigations into the glycocalyx structure and function during the lung transplant process.

## Figures and Tables

**Table 1:** Human donor EVLP data.

No.	Type	Age	Sex	Weight (kg)	Mode of death	Donation process	PaO_2_ at retrieval (mmHg)	Time of the first sample (mins)	Transplanted	Reason for declined transplant
1	Experimental	60	Female	127	Cardiac arrest	DCD	469	300	No	Worsening pulmonary oedema
2	Experimental	40	Male	90	Subarachnoid haemorrhage	Brain death	304	240	No	Bilateral consolidation
3	Experimental	63	Male	75	Asphyxia	Brain death	400	150	No	Age, heavy smoking history, and emphysematous bullae at retrieval
4	Experimental	40	Female	50	Cerebral haemorrhage	Brain death	250 (PEEP 10)	0	No	Blood group B, poor gas exchange, and heavy smoker
5	Clinical	N/A	Male	N/A	Brain injury from fall	Brain death	228	0	No	Poor gas exchange and long cold ischaemic time
6	Clinical	56	Female	90	Subarachnoid haemorrhage	Brain death	181	0	Yes	
7	Clinical	30	Female	N/A	Anoxic brain injury	Brain death	266	0	Yes	
8	Clinical	53	Male	90	Cerebral hypoxia after cardiac arrest	DCD	N/A	0	No	Poor gas exchange
9	Clinical	26	Female	N/A	Traumatic brain injury from fall	Brain death	366	0	Yes	

DCD: donation after circulatory death; brain death: donation after brain death; PaO_2_: arterial oxygen concentration.

## Data Availability

Public availability of some of the human data used to support the findings of this study is restricted by The Prince Charles Hospital Human Research and Ethics Committee in order to protect patient confidentiality. The porcine data used to support the findings of this study are included within the article, and additional data is available from the authors upon request.

## Supplementary Materials

Supplementary Materials Supplementary Method 1: retrieval protocol for human lungs and human EVLP. The protocol for retrieval of human organs and the establishment of them on EVLP. It includes EVLP setup, perfusion strategies, and acceptance criteria. Supplementary Method 2: retrieval protocol for porcine lungs and porcine EVLP. The protocol for the porcine anaesthetic developed by the team at Queensland Lung Transplant Service, The Prince Charles Hospital. It includes lung flush, retrieval, and storage techniques. Also detailed are the setup of the EVLP circuit and establishment of porcine lungs on the EVLP circuit. Supplementary Method 3: porcine EVLP perfusion and ventilation strategies in resuscitation and evaluation mode on Vivoline LS1. Perfusate gas supply indicates the gas(es) supplied to the oxygenator/deoxygenator unit during the two phases. It also describes the initial perfusion technique followed by the maintenance of perfusion strategies, including perfusion flow rates and pulmonary artery pressures. Additionally, the ventilation strategies including the tidal volume, inspiratory pressure, positive end-expiratory pressure, respiratory rate, and recruitment procedures. Supplementary Method 4: porcine sidestream dark field imaging technique. An in-depth description of the protocol and technique.
