# Supplementary material for: Endothelial Glycocalyx Shedding Occurs during Ex Vivo Lung Perfusion: A Pilot Study
Source: J Transplant. 2019 Aug 25;2019:6748242. doi: 10.1155/2019/6748242 (PMC6732651; doi:10.1155/2019/6748242)
Supplement: Supplementary Materials — Supplementary Method 1: retrieval protocol for human lungs and human EVLP. The protocol for retrieval of human organs and the establishment of them on EVLP. It includes EVLP setup, perfusion strategies, and acceptance criteria. Supplementary Method 2: retrieval protocol for porcine lungs and porcine EVLP. The protocol for the porcine anaesthetic developed by the team at Queensland Lung Transplant Service, The Prince Charles Hospital. It includes lung flush, retrieval, and storage techniques. Also detailed are the setup of the EVLP circuit and establishment of porcine lungs on the EVLP circuit. Supplementary Method 3: porcine EVLP perfusion and ventilation strategies in resuscitation and evaluation mode on Vivoline LS1. Perfusate gas supply indicates the gas(es) supplied to the oxygenator/deoxygenator unit during the two phases. It also describes the initial perfusion technique followed by the maintenance of perfusion strategies, including perfusion flow rates and pulmonary artery pressures. Additionally, the ventilation strategies including the tidal volume, inspiratory pressure, positive end-expiratory pressure, respiratory rate, and recruitment procedures. Supplementary Method 4: porcine sidestream dark field imaging technique. An in-depth description of the protocol and technique. [file 6748242.f1.docx]

## Supplementary Methods:

### Supplementary methods 1. Retrieval protocol for human lungs and human EVLP

Lungs were retrieved from donors in accordance with established protocols for The Prince Charles Hospital, Chermside, Australia. Following midline sternotomy, the lungs and pleura undergo visual inspection. The pericardium is opened and the pulmonary artery is cannulated (EPOA Arterial cannula, Medtronic, USA). In brain death donors, they are systemically heparinized. Following this a bolus of chlorpromazine is given and once systemic blood pressure decreases, the aortic cross clamp is place. The left atria was incised and the lungs perfused with 5.6L of ice cold Perfadex solution at a pressure of 30cmH^2^O. Once perfused, organs are inflated with air and stored in Perfadex solution at 4^O^c until transport to The Prince Charles Hospital.

The ex-vivo lung perfusion circuit (Vivoline LS1, Vivoline Medical, Sweden) was primed and calibrated as per manufacturer’s instructions. Briefly, a sterile kit was place on the rig and filled with evaluation solution (4 x500ml Steen Solution). 10,000IU heparin were added along with 2 units of CMV –ve, irradiated blood group O-ve packed red cells (450ml). The haematocrit is then checked to ensure it is 10-15%. The machine is then placed in the priming phase and the pressure sensors are calibrated. Following this 100mg vancomycin (DBL Hospira, Australia), 200mg imipenem (Merck Sharp & Dohme, Australia) was added to the system. The temperature set for 14 ^O^c and flow started at 2L/min to mix the evaluation solution components for a minimum of 15minutes. The pH of the solution is then corrected with THAM (1ml for every -1 in the base excess). During all phases of perfusion, the evaluation solution is treated with a gas blend of nitrogen (93%) and carbon dioxide (7%) at 10L per minute. In the priming and reconditioning phase, the perfusate is also oxygenated with 2.7L/min O_2_.

The lungs are then connected to the EVLP circuit via a pulmonary artery cannula and by insertion of a suitable sized tracheal tube into the trachea. The temperature probe and sampling catheter are then sutured into the left atrial remnant. The various catheters area attached to the circuit and the tracheal tube, via the EVLP circuit housing, is attached to a ventilator. The VivoLine LS1 is then changed into reconditioning mode with flow started at 0.2L/hr with the shunt, an opening immediately before the pulmonary artery cannula, in the open position to allow perfusion of the evaluation solution to displace the air in the circulatory system and pulmonary artery. When all the air is gone the flow is increased to the desired maximum flow rate and temperature of 37 ^O^c. During the priming, reconditioning and evaluation phase the system is both flow and pressure limited, this means that the set value that is reached first (pulmonary artery flow rate 70ml/kg; Pulmonary artery pressure 20mmHg) will limit the other parameter. Usually this means the flow will be initially limited by the pulmonary pressure, then as the lungs warm and the pulmonary vascular resistance decreases, the flow rate increases. Once perfusate temperature reaches 25 ^O^C the shunt is closed with ventilation started once the lungs reach 32 ^O^C. The initial ventilator settings are a tidal volume of 8ml/kg, respiratory rate 12 breathe per minute, PEEP 5cmH2O and an FiO21%.

Once the lungs reach 36 ^O^C, the Vivoline is then changed to evaluation mode and the oxygen supply to the system is disconnected. The ventilation FiO2 is changed from air to 100% oxygen. After 10 minutes of perfusion the lung perfusate emerging from the left atrium is sampled on a blood gas analysis. Lungs were considered suitable for transplant if all the following criteria were reached: (1) PaO2 >300mmHg on FiO2 100% at two consecutive time points within the 4hr maximum period of EVLP; (2) stability or improvement of other lung function parameters during EVLP including pulmonary vascular resistance, compliance and airway pressures; (3) achieving full flow in the circuit (70ml/kg/min) at normal pulmonary artery pressures; (4) normal collapse tests; (5) surgeon clinically satisfied with lung evaluation.

### Supplementary methods 2. Retrieval protocol for Porcine lungs and Porcine EVLP

Four landrace x large white pigs, weighing 40-48kg, were pre-medicated with a combination of zolazepam/tiletamine (Zoletil. Virbac, Australia) 5mg/kg and butorphanol 0.15mg/kg (Torbugesic. Zoetis, Australia) intramuscularly and solumedrol (Solumedrol, Pfizer, Australia) 1 gram intravenous (IV). Anesthesia was achieved with IV propofol (Propofol-Lipuro 1%, B. Braun, Germany) followed by oral intubation with cuffed tube. Anesthesia was maintained using isoflurane 5% (Isoflo. Abbott Australasia, Australia) through an in-line vaporizer with ventilation on 100% oxygen maintained via a volume-controlled anesthetic ventilator (EV500, Ulco Medical, Australia) with a tidal volume of 8ml/kg, positive end expiratory pressure (PEEP) of 5cm H^2^O and a respiratory rate at of 18 breathes/ minute varied to maintain CO^2^ between 35-45mmHg.

Under sterile conditions a median sternotomy was then performed, the pericardium opened followed by opening of the pleural cavities to visually inspect the lungs. Heparin 500IU/kg (Pfizer, Australia) was then administered into the left atrial appendage and an atrial blood gas was collected followed by 2x 450ml bags of whole anticoagulated blood (Bloodpack, Fenwal, USA). Once removed the blood preparation technique involved the 2x 450ml bags of whole anticoagulated blood being centrifuged at 3200 RPM for 15mins at 4^O^c (J6-M1 Beckman Coulter centrifuge) and serum with the buffy coat removed using simple decanting. After lung inspection, the pulmonary artery was then cannulated (EPOA Arterial cannula, Medtronic, USA) and secured in place with a suture. Once this line was primed to remove any air a bolus of pentobarbital (Virbac, Australia) (0.5ml/Kg) was administered intra-cardiac into the left ventricle to induce cardiac arrest. The left atria was incised along with the inferior vena cave followed immediately by the commencement of ice cold Perfadex (Xvivo Perfusion AB, Sweden) 60ml/kg via the pulmonary artery at 20cmH^2^O pressure. A 2.8L bag of Perfadex was primed with 30mg Glyceryl Trinitrate (Hospira, Germany), 25000IU heparin (Pfizer, Australia), 360mg THAM (Addex-THAM, Fresenius Kabi AB, Sweden), 130mg Calcium Chloride (Phebra, Australia). The lungs were recruited with normal air and insufflated to 20cm H^2^0 whilst the heart/lung block was removed from the chest cavity and placed in a sterile bag (Defries Industries Pty Ltd, Victoria, Australia) with 1L Perfadex and sealed, before being double bagged with ice cold Plasmalyte (Baxter, Australia) being added to the outer bag before being placed on ice for cold storage at 4^O^c.

After 4hrs (+/-30mins) of cold storage the lungs were removed from the bag and the heart was excised on the back table. The lungs were then attached to the EVLP rig (Vivoline LS1, Vivoline medical, Sweden) via cannulation of the pulmonary artery and the trachea. Perfusate set-up and EVLP circuit preparation was similar to the human protocol (Supplementary methods 1), except for the use of autologous porcine blood. Perfusion was commenced in resuscitation mode at 14^O^C, at 0.1L/min with the shunt open to allow de-airing, similar to the protocol in human EVLP. Over the first hour, the flow rate was gradually increased (see Supplementary Methods 3) similar to that described by Cypel et al .(1), whilst maintaining pulmonary artery pressure < 20mmHg. This is an automated process in resuscitation mode on the Vivoline SL1, however the perfusion rate and temperature were manually increased at a slower rate than automated to prevent rapid warming of the lungs. After 20mins, the shunt was closed when the pulmonary artery pressure remained below 20mmHg.

Once the lungs reached 32^O^C, usually after 30-40mins, ventilation was commenced with a pressure-controlled ventilator (Puritan Bennett 840 Ventilator, Covidien, USA) as described in the table in supplementary method 3. Recruitment maneuvers were carried out hourly with two inspiratory holds with increased peak airway pressure to 25cm H_2_O held for 10seconds, whilst flow was slowed. PEEP was increased over time on EVLP in accordance with amount of visible pulmonary edema. In evaluation mode flow rate was set at to 40% of maximum cardiac output (maximum cardiac output= 100ml/kg). Perfusion and ventilation were continued until lungs were either deemed too oedematous, with perfusate clearly accumulating in the tracheal tube, or 12hrs had elapsed. Control 4 which only had FiO_2_ 21% available, had a much higher pulmonary vascular resistance compared to the other controls. To reduce PVR, we used 10ml glycerol trinitrate (Hospira, Germany) and 1mg milrinone (Sanofi, Australia) at 4 and 6hrs, respectively which gave a temporary response.

### Supplementary Methods 3. Porcine EVLP perfusion and ventilation strategies in resuscitation and evaluation mode on the Vivoline SL1. Perfusate gas supply indicates the gas(s) supplied to the oxygenator/ deoxygenator unit during the two phases.

|  | Resuscitation | Evaluation |
| --- | --- | --- |
| Perfusion |  |  |
| starting temp | 14 | 32 |
| completion temp | 32 | 36 |
| Flow rate | Initially 0.2L/min  10mins 0.2L/min  20mins 20% max flow  30mins 30% max flow  40mins 50% max flow  50mins 80% max flow | max flow = 40ml/kg/min |
| Pulmonary artery pressure | <20mmHg | <20mmHg |
| Perfusate Oxygen supply | 2.7L/min | 0L/min |
| Perfusate N2/CO2 supply | 10L/min | 10L/min |
| Ventilation |  |  |
| Respiratory rate |  | 8 min |
| PEEP |  | 5cmH^2^O to 10cmH^2^O |
| Tidal volume |  | 6ml/kg |
| FiO^2^ |  | 100% |

### Supplementary Methods 4. Porcine Side stream dark field imaging technique

The exposed surface of the lung, with its adherent visceral pleura, was cleaned of blood using normal saline soaked swabs. By applying glycerol 10% to the pleural surface of the lung, pleural vessel imaging clarity was enhanced. A sterile disposable tip (Microscan BV, Amsterdam, Netherland) was used to cover the tip of the microscope, which was then placed perpendicularly on the surface of the lung after complete expiration while the lung was stable. The microscope was placed in manual mode, and once manually focused, stability and light intensity parameters were met, recording was started. Images were captured with 5x magnification with a 0.2NA providing 325x on screen magnification. The frame rate was 30/second, 1-3 seconds of video of was recorded (640x480pixels) using Automated Vascular Analysis software (Microvision Medical BV, Amsterdam, Netherland) and later analyzed. A series of videos (minimum 3 areas of pleural vasculature) were collected at the upper lobe of the porcine lungs in situ. The same process was repeated once lungs were stable on the EVLP circuit and being fully ventilated at the 1, 6 and 12hr (or completion) time points.

References:

1. Cypel M, Yeung JC, Hirayama S, Rubacha M, Fischer S, et al. Technique for prolonged normothermic ex vivo lung perfusion. J Heart Lung Transplant 2008:27:1319-1325.
